# Knockdown of CREB3/Luman by shRNA in Mouse Granulosa Cells Results in Decreased Estradiol and Progesterone Synthesis and Promotes Cell Proliferation

**DOI:** 10.1371/journal.pone.0168246

**Published:** 2016-12-14

**Authors:** Fan Zhao, Nan Wang, Yanglei Yi, Pengfei Lin, Keqiong Tang, Aihua Wang, Yaping Jin

**Affiliations:** 1 Key Laboratory of Animal Biotechnology of the Ministry of Agriculture, Northwest A&F University, Yangling, Shaanxi, China; 2 College of Veterinary Medicine, Northwest A&F University, Yangling, Shaanxi, China; 3 College of Animal Science and Technology, Northwest A&F University, Yangling, Shaanxi, China; China Agricultural University, CHINA

## Abstract

Luman (also known as LZIP or CREB3) is a transcription factor and a member of the cAMP responsive element-binding (CREB) family proteins. Although Luman has been detected in apoptotic granulosa cells and disorganized atretic bodies, the physiological function of Luman in follicular development has not been reported. Our objective is to determine the role of Luman in folliculogenesis by knocking down Luman expression in mouse GCs (granulosa cells) using shRNA. Luman expression was successfully knocked down in mouse GCs at the mRNA and protein level, as confirmed by real-time quantitative PCR, western blot and immunofluorescence staining, respectively. Knockdown of Luman significantly decreased the concentrations of estradiol (E2) and progesterone (P4) in cell culture medium. Furthermore, Luman knockdown promoted cell proliferation but had no effect on cell apoptosis. To elucidate the regulatory mechanism underlying the effects of Luman knockdown on steroid synthesis and cell cycle, we measured the mRNA and protein expression levels of several related genes. The expression of Star, Cyp19a1, and Cyp1b1, which encode steroidogenic enzymes, was down-regulated, while that of Cyp11a1 and Runx2, which also encode steroidogenic enzymes, was up-regulated. The expression of the cell cycle factors Cyclin A1, Cyclin B1, Cyclin D2, and Cyclin E was significantly up-regulated. Among apoptosis-related genes, only Bcl-2 was down-regulated, while Caspase 3, Bax and p53 were not significantly affected, suggesting that Luman knockdown may regulate cell cycle activity and hormone secretion at the transcriptional and translational level in mouse GCs. The expression of two important genes associated with folliculogenesis in mouse GCs, Has2 and Ptgs2, were also significantly altered by Luman knockdown. In conclusion, the findings of this study indicate that Luman regulates mouse GCs modulation of steroid synthesis, cell cycle activity and other regulators of folliculogenesis.

## Introduction

Follicles are the basic functional units of mammalian ovaries. In rodents, follicular development begins during the neonatal period when primordial follicles form. Following an initial growth period, activated primordial follicles, which are bordered by a single layer of flattened granulosa cells (GCs) and surround the primordial oocyte, develop into primary, secondary, and eventually antral follicles [1]. During this process, follicular growth is facilitated by GC proliferation and follicular fluid formation. Further development entails GCs and follicular tissue cyto-differentiation. However, only a few follicles successfully complete the cyto-differentiation process, as most GCs die by apoptosis [2], a programmed cell death mechanism that ensures ovulation of only the most fertilizable oocytes.

Local regulatory systems play an important role in governing the timing of folliculogenesis and in determining whether a follicle becomes dominant or atretic. It is well known that follicle-stimulating hormone (FSH) and luteinizing hormone (LH) can be sensed by GCs and affect the early stages of folliculogenesis. GCs have many regulatory functions, as they produce steroids and promote oocyte growth [3,4]. Follicular development and atresia can also be regulated by crosstalk involving cell death and survival signals, including signals conveyed by endocrine hormones and intraovarian regulators [5].

Luman (also called LZIP or CREB3) is a member of CREB3 (cAMP responsive element-binding) subfamily of the basic leucine-zipper (bZIP) transcription factors [6]. When inactivated, Luman is a transmembrane protein with the N-terminus facing the cytoplasm and the C-terminus penetrating through the endoplasmic reticulum (ER) membrane [7]. With such a structural property, Luman can be rapidly activated through regulated intramembrane proteolysis (RIP) in response to ER stress [8]. After activation, it will be transported from the ER to the Golgi apparatus and sequentially been cleaved to release the N-terminal fragment[9]. The released N-terminus, which encodes the transcription activation domain and the bZIP region, translocate to the nucleus to activate the target genes. The known candidate genes regulated by Luman include homocysteine-induced ER protein (Herp) and ER degradation-enhancing mannosidase-like protein [7,10], which contain CREs and unfolded protein response elements (UPREs) [6,11]. It is thought that the interaction between Luman and host cell factor C1 plays a role in the establishment of latency during herpes simplex virus (HSV) infection. This protein also plays a role in leukocyte migration[12–15], tumor suppression[16], and dendritic cell maturation [17,18]. Luman expression in sensory neurons [11,19] is indicative of its potential role in the inhibition of astrocyte differentiation [20].

The CREB3 family members isolated from mouse and human are closely related to *Drosophila* dCREB-A/BBF2. They share considerable homology within the transmembrane domain, the ER luminal domain, and bZIP domain that mediates DNA binding and dimerization [21]. Rose *et al*. [22] reported that dCREB-A/BBF2 is necessary for *Drosophila* embryonic development, suggesting the possible role of CREB3 protein in reproduction. Previous work in our lab found that Luman protein was detected in the luminal, glandular epithelium, and decidual cells during mouse implantation and decidualization [23]. Luman also abundantly expressed in mouse ovarian GCs, irrespective of follicular maturation [24]. However, little is known regarding the role of Luman in follicular growth and development. The present work sought to determine the role of Luman in folliculogenesis.

## Materials and Methods

### Animals

Immature female Kunming white mice (SPF grade, 21 days old) were purchased from the Experimental Animal Center of the Fourth Military Medical University. All mice were fed chow and water daily and housed under light-controlled conditions (12:12-hour light-dark cycles) at 22°C. All procedures were approved by the Committee for Ethical Animal Care and Experimentation of Northwest A&F University.

### Mouse GC isolation and culture

Female mice were injected intraperitoneally with pregnant mare serum gonadotropin (10 IU per mouse; Sigma, St. Louis, MO, USA) to facilitate GC proliferation. After 44 hours, the animals were sacrificed by decapitation, and their ovaries were quickly removed and placed in DMEM/F12 (1:1, HyClone) supplemented with penicillin (100 U/mL, Sigma, St. Louis, MO, USA) and streptomycin (100 mg/mL, Sigma, St. Louis, MO, USA). GCs were harvested by puncturing individual ovarian follicles with a 27-gauge needle attached to a 1 mL syringe, collected by centrifugation (3000 rpm, 3 min) and cultured in a humidified incubator at 37°C with 5% CO_2_ in DMEM/F12/PS for 48 h before treatment.

### Transfection of mouse GCs with shLuman lentiviruses

shLuman lentiviruses were packaged according to the method described previously by Chen *et al*. [25]. Lentivirus vectors encoding *Luman* shRNA (shLuman) and a non-silencing negative control (shRNA-negative) were constructed by our group. The sequence of shRNA-negative is 5’-GATCCGATGAAATGGGTAAGTACATTCAAGAGATGTACTTACCCATTTCATCTTTTTTG-3’, and the sequence of shLuman is 5’- GATCCACAGGAGATGTCTAGGCTGATTTCAAGAGAATCAGCCTAGACATCTCCTGTTTTTTTG-3’. After being filtered through a 0.45 μm filter (Millipore), the cell supernatant (shLuman lentivirus and shRNA-negative lentivirus) was stored at -80°C. The day before transfection, 2 × 10^5^ GCs were seeded in 6-well plates at 60–70% confluence. Complete culture medium was replaced by a solution containing 2 mL of shRNA-negative or shLuman (2 × 10^−8^ TU/mL) with 2 μL (8 mg/mL) of polybrene (Genechem). After twelve hours, the lentivirus solution was replaced by complete culture medium and cultured for 48 h. Cells were subsequently collected for various experiments.

### Confocal immunofluorescence microscopy

Mouse GCs were fixed in 4% formaldehyde for 30 min and permeabilized in 0.2% Triton X-100 for 15 min at room temperature. After being blocked with 1% BSA in PBS, cover slips were incubated with an anti-Luman primary antibody (1:200 dilution; prepared by our laboratory), followed by incubation with an Alexa594-conjugated anti-mouse secondary antibody (1:300 dilution; Invitrogen, A31572) for 1 h at 37°C. Nuclei were stained with 4′,6-diamidino-2-phenylinole (DAPI) (Beyotime Co. Ltd) for 10 min. Images were captured with a digital camera under a Nikon epifluorescence microscope (Eclipse 80; Nikon, Tokyo, Japan).

### Real-time quantitative PCR

Total RNA was isolated from at least 6 × 10^5^ cells with TRIzol reagent (TaKaRa Bio, Inc.), and RNA quantity and purity were determined using a NanoDrop spectrophotometer. One microgram of total RNA was treated with DNase-I (TaKaRa Bio, Inc.), and cDNA was synthesized using a PrimeScript^TM^ RT reagent Kit (TaKaRa Bio, Inc.). qRT-PCR was performed on a QuantStudio 6 Flex Real-time PCR system (Thermo Fisher Scientific Inc., USA). Each reaction was performed in a 20 μL reaction volume containing 10 μL of 2×SYBR^®^ Premix Ex Taq^TM^Ⅱ (Tli RNaseH Plus), 0.8 μL of ROX Reference Dye II (50×) (TaKaRa Bio, Inc.), 1.4 μL of cDNA (35 ng total RNA), 0.8 μL of each primer (10 μM) and 6.2 μL of nuclease-free water. The PCR cycling conditions were as follows: one cycle at 95°C for 30 s, followed by 40 cycles at 95°C for 5 s and 60°C for 30 s. A melting curve analysis was performed at the end of each PCR programme to exclude the formation of nonspecific products. Amplification was conducted to analyze relative mRNA expression levels. Primer pairs and their respective annealing temperatures are presented in Table 1. *β-actin* served as a reference gene. At least three biological replicates were performed for each sample, and the fold-changes in the relative quantities of the amplified targets were calculated by the 2^-△△Ct^ method.

**Table 1:** Primer sequences used for real-time quantitative PCR (RT-qPCR)

Gene	GenBank Accession no	Forward (5′-3′)	Reverse (5′-3′)
*β-actin*	NM_007393	GCAAGCAGGAGTACGATGAG	CCATGCCAATGTT GTCTCTT
*Luman*	NM_006368	CTTCTCCGACTCCAACCTTC	CCACATCCTCACACCTAACC
*Star*	NM_011485.4	CTTGGCTGCTCAGTATTGAC	TGGTGGACAGTCCTTAACAC
*Cyp19a1*	NM_007810.3	GACACATCATGCTGGACACC	CAAGTCCTTGACGGATCGTT
*Cyplbl*	NM_009994.1	CACTATTACGGACATCTTCGG	AGGTTGGGCTGGTCACTC
*Cyp11a1*	NM_019779.3	CGATACTCTTCTCATGCGAG	CTTTCTTCCAGGCATCTGAAC
*Runx2*	NC_000083.6	TACCTGCCATCACTGACGTG	CTGGCGGGGTGTAGGTAAAG
*Cyclin A1*	Z26580.1	GCCTTCACCATTCATGTGGAT	TTGCTGCGGGTAAAGAGACAG
*Cyclin B1*	NM_172301.3	AAGGTGCCTGTGTGTGAACC	GTCAGCCCCATCATCTGCG
*Cyclin D2*	NM_009829.3	ACACCGACAACTCTGTGAAGC	GCCAGGTTCCACTTCAGCTTA
*Cyclin E*	NM_007633	GTGGCTCCGACCTTTCAGTC	CACAGTCTTGTCAATCTTGGCA
*Caspase-3*	NM_001284409.1	TGACTGGAAAGCCGAAACTC	GCAAGCCATCTCCTCATCAG
*Bcl-2*	NM_009741.4	CGAGAAGAAGAGGGAATCACAGG	AATCCGTAGGAATCCCAACC
*Bax*	NM_007527.3	AGGATGCGTCCACCAAGAA	CAAAGTAGAAGAGGGCAACCAC
*p53*	AB020317.1	TACAAGAAGTCACAGCACAT	GATAGGTCGGCGGTTCAT
*Has2*	NM_008216.3	ACCCTGCCTCATCTGTGGAGA	TGTTGGTAAGGTGCCTGTCGT
*Ptgs2*	NM_011198.3	CTCTATCACTGGCACCCCCTG	GAAGCGTTTGCGGTACTCATT

### Western blot analysis

Mouse GCs were rapidly washed with ice-cold PBS. The lysate was separated from cellular debris via centrifugation at 13,000 rpm for 10 min. After total protein was measured by BCA assay (Nanjing KeyGen Biotech Co., Ltd., Nanjing, China), the samples were stored at −80°C for subsequent use. For SDS-PAGE, the protein samples were separated on 12% polyacrylamide gel and transferred to PVDF membranes (Millipore, Bedford, MA). The membranes were blocked with 10% skim milk diluted in PBS and supplemented with 0.05% Tween-20 and incubated overnight at 4°C with the following primary antibodies: anti-Luman (1:400, made by our laboratory), anti-Cyp19 (1:500, Santa Cruz, USA), anti-Cyp11a1 (1:500, Santa Cruz, USA), anti-Star (1:500, Santa Cruz, USA), anti-Runx2 (1:500, Santa Cruz, USA), anti-Caspase 3 (1:500, Santa Cruz, USA), anti-Bcl-2 (1:500, Santa Cruz, USA) and anti-β-actin (1:1000, Tianjing Sanjian Biotech Co., Ltd., Tianjing, China). Then, following three washes with PBS containing 0.1% Tween 20, the membranes were incubated with the corresponding secondary antibody conjugated to HRP (1:2000, Zhongshan Golden Bridge Biotechnology, Nanjing, China) for 1 h at room temperature. Finally, bands were visualized using a gel imaging system (Tannon Science & Technology Co. Ltd., Shanghai, China) and then digitized using Quantity One software (Bio-Rad Laboratories, Hercules, CA, USA).

### Estradiol and progesterone measurements

After 48 h of shLuman lentivirus transfection, the cells were counted. Then, GC serum-free culture medium was used to measure estradiol and progesterone concentrations with mouse estradiol (E_2_) and progesterone (P_4_) ELISA kits, respectively (Ji Yin Mei, Co. Ltd., Wuhan, China). The test procedures were performed according to the manufacturer's instructions.

### Cell cycle analysis

After being harvested, transfected GCs (2 × 10^5^ cells/well in 6-well plate) were washed with PBS and fixed in ice-cold 70% ethanol overnight at 4°C. Then, the cells were stained with propidium iodide/RNase A solution at 37°C for 30 min in a dark chamber. Flow cytometric analyses were conducted using a BD FACSCalibur system and ModFit LT for Mac V3.0 software. For each determination, a minimum of 20,000 cells was analyzed. All experiments were repeated three times.

### Cell apoptosis detection

GC apoptosis was detected via dual-staining with Annexin V-APC and 7-AAD (Annexin V-APC/7-AAD Apoptosis Detection Kit, KeyGEN Co. Ltd., Nanjing, China). The procedure was performed according to the manufacturer’s instructions with some modification. Briefly, transfected cells (2 × 10^5^ cells per 6-well plate) were harvested by digestion with trypsin without EDTA at 37°C for 20 min and centrifuged at 500 × g for 5 min. Then, the cell pellets were washed and re-suspended in PBS twice. Apoptotic cells were stained with the 7-AAD and Annexin V-PE staining solution provided by the kit and detected using a fluorescent activated cell sorter (FACS) (Becton, Dickinson and Company, USA). Experiments were repeated at least three times.

### Statistical analysis

Data were analyzed via one-way ANOVA, followed by Fisher’s least significant difference test (Fisher’s LSD) and an independent-samples T test with SPSS (Statistical Package for the Social Sciences) software (Version 13.0; SPSS, Inc., Chicago, IL. USA). *P* < 0.05 was considered significant. All data are represented as the mean ± SEM of repeated experiments (n = 3).

## Results

### Luman was efficiently knocked down by shLuman

**Fig 1:**
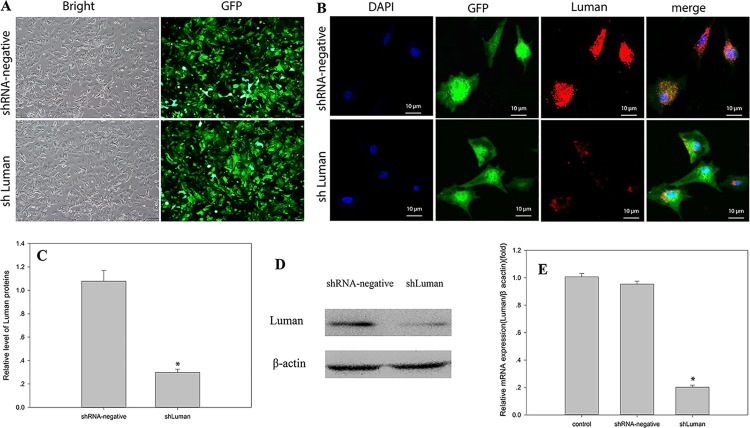
Transfection and knockdown efficiency of shLuman on mouse GCs. (A) Transfection efficiency was observed by fluorescence microscopy in the shRNA-negative and shLuman transfected primary mouse GCs. (B) The Luman protein expression was observed via immunofluorescence staining in primary mouse GCs. (C~D) Luman protein expression level was detected by western blotting in the shRNA-negative and shLuman groups. (E) *Luman* mRNA expression level was detected by real-time PCR in the shRNA-negative and shLuman groups. Non-infected primary mouse GCs were shown as control. The levels of mRNA were normalized to that of *β-actin*. Values are presented as the mean ± SEM, n = 3. Asterisks indicate significant differences (*P* < 0.05).

In order to determine the efficiency of shLuman vector, mouse GCs were transfected with pCD513B-U6-shLuman lentivirus. The expression of GFP was observed under fluorescent microscopy at 48 h after transfection. As shown in Fig 1A, around 90% of the GCs were transfected by the lentivirus. The protein level of Luman was detected by immunofluorescence staining (Fig 1B) and western blotting (Fig 1C and 1D), respectively. The Luman protein expression was significantly decreased by shLuman lentivirus. The mRNA level of Luman was detected by real-time PCR (Fig 1E), which showed a 80% decrease. These data indicated that the pCD513B-U6-shLuman lentivirus efficiently knocked down Luman expression at both transcriptional and translational levels.

### Luman knockdown reduced the concentrations of estradiol and progesterone in mouse GC culture medium

**Fig 2:**
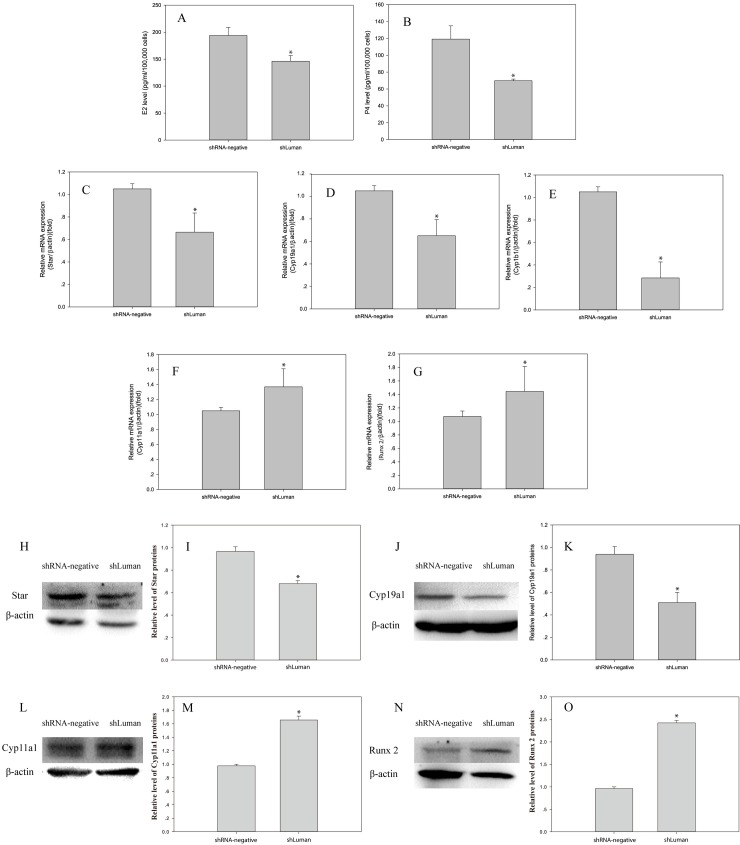
Effects of Luman knockdown via shLuman lentivirus transfection on estradiol (E2) and progesterone (P4) secretion in mouse GCs. (A~B) Concentrations of estradiol (E2) and progesterone (P4) in mouse GC culture medium after shLuman lentivirus transfection compared with shRNA-negative lentivirus transfection at 48 h. (C~O) The mRNA and protein levels of genes (*Star*, *Cyp19a1*, *Cyp1b1*, *Cyp11a1* and *Runx2*) associated with hormonal secretion were compared between the shLuman and shRNA-negative groups. The levels of mRNA were normalized to that of *β-actin*. Values are presented as the mean ± SEM, n = 3. Asterisks indicate significant differences (P < 0.05).

To assess the effect of silencing Luman on steroid hormone levels, we measured the concentrations of estradiol (E_2_) and progesterone (P_4_) in culture medium at 48 h post-transfection. The results showed that after transfection, the levels of estradiol and progesterone were significantly lower in the shLuman group (*P* < 0.01 and *P* < 0.05, respectively) than in the shRNA-negative group (Fig 2A and 2B). We further analyzed the mRNA and protein expression of several genes encoding steroidogenic enzymes, including *Star*, *Cyp19a1*, *Cyp1b1*, *Cyp11a1* and *Runx2*. The results showed that Luman knockdown significantly decreased the *Star* (regulates cholesterol transport), *Cyp19a1* and *Cyp1b1*, while it increased the *Cyp11a1* and *Runx2* (Fig 2C–2O) at both transcriptional and translational level. These results confirm that Luman is involved in the regulation of steroidogenesis in mouse GCs.

### Luman knockdown altered mouse GC growth and proliferation

The effect of Luman depletion on cell cycle was investigated by flow cytometry. GC nuclear contents were stained with propidium iodide (PI). Then, the stained cells were subjected to FACS analysis. The results are shown in Table 2. Compared with the control group, Luman-depleted GCs exhibited a significantly (*P* < 0.05) increased cell population at G1 phase, as well as a decreased cell population at S phase and G2 phase. The percentage of cells in G1 phase increased from 74.73% in shRNA-negative cells to 85.67% in shLuman cells.

**Table 2:** Analysis of mouse GCs cell cycle by FACS at 48 h post-trasnsfection with a shLuman lentivirus and a shRNA-negative lentivirus (mean ± SEM, n = 3).

	G1 (%)	S (%)	G2 (%)
shRNA-negative group	74.73%±0.51	14.53%±0.28	10.73%±0.29
shLuman group	85.67%±0.65*	10.32%±0.45*	4.01%±0.22*

All results were evaluated by one-way ANOVA.

* An asterisk indicates the level of significance within the columns (P < 0.05).

**Fig 3:**
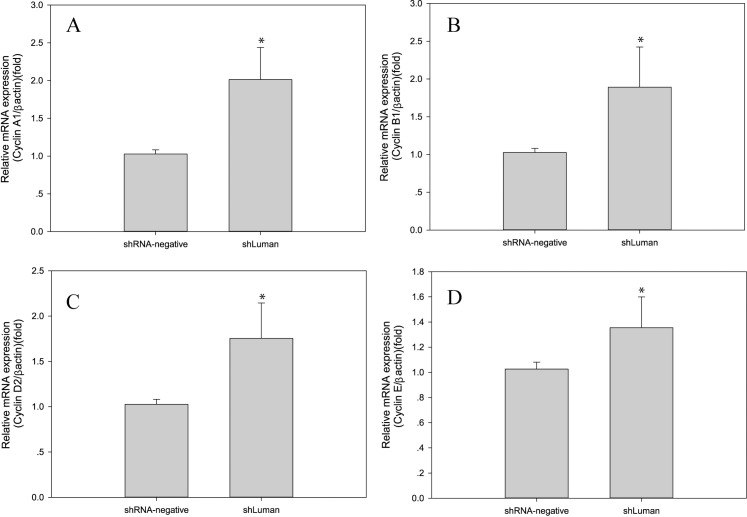
Effects of Luman knockdown on cell cycle. (A~D) The mRNA expression levels of the indicated cell cycle-related genes (*Cyclin A1*, *Cyclin B1*, *Cyclin D2* and *Cyclin E*) were increased in the shLuman group compared with the shRNA-negative group. The levels of mRNA were normalized to that of *β-actin*. Values are presented as the mean ± SEM, n = 3. Asterisks indicate significant differences (P < 0.05)

The mRNA levels of the indicated cell cycle factors (*Cyclin A1*, *Cyclin B1*, *Cyclin D2* and *Cyclin E*) were determined by real-time PCR. The results showed that after shLuman silencing, significant increases in the levels of *Cyclin A1*, *Cyclin B1*, *Cyclin D2* and *Cyclin E* mRNA expression were observed (*P* < 0.05 or *P* < 0.01) (Fig 3A–3D). These results indicate that Luman plays a crucial role in GC growth.

### Effects of Luman knockdown on mouse GC apoptosis

To determine the role of Luman in GC apoptosis regulation, we detected apoptotic cells using a flow cytometry-based apoptosis detection kit. The results showed that Luman down-regulation did not significantly affect the number of cells that underwent apoptosis in the shLuman group compared to the shRNA-negative group (Table 3).

**Table 3:** Measurement of cell apoptosis by FACS at 48 h post transfection with the shLuman lentivirus and the shRNA-negative lentivirus (mean ± SEM, n = 3).

	Live cells (%)	Apoptpsis cells (%)
shRNA-negative group	89.3%±0.6	10.7%±0.6
shLuman group	87.9%±0.4	12.1%±0.4

**Fig 4:**
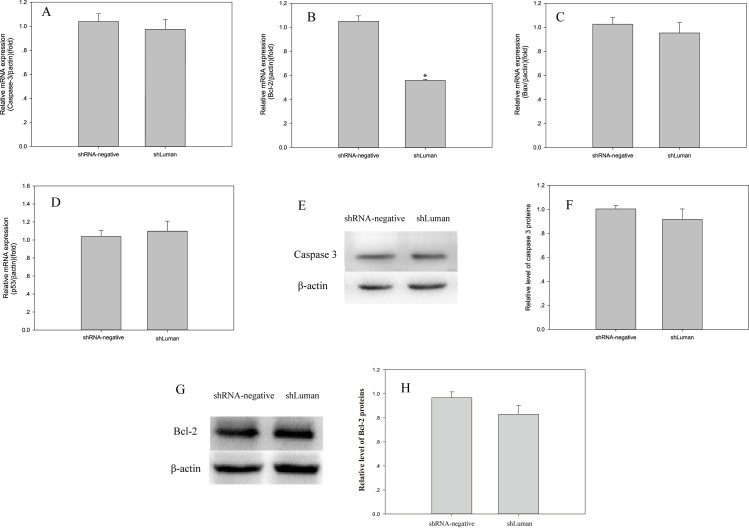
Effect of Luman knockdown on cell apoptosis. (A~D) Relative mRNA expression levels of genes (*Caspase 3*, *Bcl-2*, *Bax* and *p53*) associated with cell apoptosis were compared between the shLuman and shRNA-negative groups. The levels of mRNA were normalized to that of *β-actin*. (E~H) Caspase 3 and Bcl-2 protein expression levels in mouse GCs were detected by western blotting in the shRNA-negative and shLuman groups. Values are presented as the mean ± SEM, n = 3. Asterisks indicate significant differences (P < 0.05).

To further elucidate the effects of Luman knockdown on apoptosis, we quantified the cellular mRNA expression levels of apoptotic inducers, such as *Caspase 3 and p53*, and those of *Bcl-2* family members. As shown in Fig 4B, Luman knockdown significantly reduced the level of *Bcl-2* mRNA expression. However, no significant differences in *Caspase 3*, *Bax* and *p53* mRNA expression were observed (Fig 4A, 4C and 4D). Western blotting results indicated that there was no significantly difference of Caspase 3 protein expression between the shLuman and shRNA-negative group (Fig 4E and 4F). While a slight decrease of Bcl-2 protein expression was observed (Fig 4G and 4H), which was consistent with the RT-qPCR results.

### Luman knockdown altered the expression of genes associated with folliculogenesis in mouse GCs

**Fig 5:**
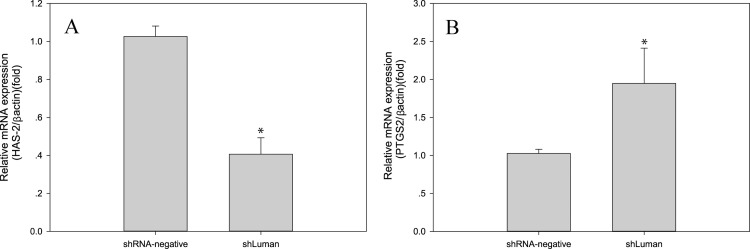
Effects of Luman knockdown on the mRNA expression levels of genes related to folliculogenesis and luteinization. (A) The mRNA expression level of *Has2* was decreased, and (B) the mRNA expression level of *Ptgs2* was increased in the shLuman group compared with the shRNA-negative group. The levels of mRNA were normalized to that of *β-actin*. Values are presented as the mean ± SEM, n = 3. Asterisks indicate significant differences (P < 0.05).

We further studied the expression profiles of *Has2 (Hyaluronan synthase 2)* and *Ptgs2 (prostaglandin-endoperoxide synthase 2)*, which are associated with mouse folliculogenesis, ovulation, and luteinization, to determine the potential involvement of Luman in mouse ovarian function. The mRNA expression level of *Has2* decreased (*P* < 0.01), while that of *Ptgs2* increased by 70% (*P* < 0.05) in mouse GCs after Luman knockdown (Fig 5), suggesting that Luman plays an important role in gene expression in the mouse ovary during folliculogenesis and ovulation.

## Discussion

Previous studies have shown that Luman is abundantly expressed in mouse ovarian GCs. However, the molecular and physiological functions of Luman in GC apoptosis, cell cycle activity, and hormonal synthesis remain poorly understood. To address these questions, we constructed a recombinant lentiviral vector, pCD513B-U6-shLuman, to silence Luman mRNA and protein expression in mouse GCs. Then, we studied the effects of Luman knockdown on GC hormone production, cell cycle activity, apoptosis, and ovulation.

There exists considerable evidence indicating that the nutritional and metabolic aspects of follicular growth are mediated by hormones and growth factors secreted by GCs [26], including estradiol (E_2_) and progesterone (P_4_) [27–29]. Lan *et al*. reported that Luman RNA and protein may be directly stimulated by estradiol to regulate embryo-uterine interactions [23]. In the present study, the concentrations of estradiol and progesterone were significantly decreased in the Luman silencing group in compared with the shRNA-negative group. The possible reason for the reductions in the levels of the two hormones could be the decreases in the *Star* (the protein associated with the transport of cholesterol across the mitochondrial membrane), *Cyp1b1* (the monooxygenase that catalyzes cholesterol and steroid synthesis), and *Cyp19a1* (the enzyme responsible for androgen aromatization to estrogen) [30]. However, the expression level of *Cyp11a1* (the rate-limiting enzyme in progesterone synthesis) was up-regulated after Luman knockdown in mouse GCs.

In addition to the decreased estradiol levels, an increased Runx2 expression was detected after Luman knockdown in mouse GCs. A similar phenomenon was also reported by Zhen *et al* [31], who observed up-regulation of Runx2 after knockdown of CEBPβ in porcine GCs. As a transcription factor, Runx2 is well known for regulating both intramembranous and endochondral bone formation, as well as osteoblast development and differentiation and chondrocyte differentiation. Park *et al*. [32] reported the high expression of Runx2 in the cumulus–oocyte complexes and GCs of periovulatory ovaries in mice and speculated that Runx2 is functionally linked to various aspects of luteal development. In human ovaries, Runx2 expression is negatively correlated with estradiol levels [33]. McNatty *et al*. suggested that decreased estradiol secretion is characteristic of an atretic follicle [34]. Therefore, we hypothesized that Luman may inhibit follicular atresia through interactions with *Runx2*, *Cyp19a1*, and *Star*.

Given the importance of cell cycle regulation in follicular development, we evaluated the GC cell cycle after Luman knockdown. Our results indicated that the G1 phase of the cell cycle was significantly increased and that the S and G2 phases were significantly decreased compared to the shRNA-negative group. Previous reports showed that Luman has tumor suppressor activity [35] and may be involved in regulating cell-cycle arrest in G0 and early G1 along with HCF in tsBN67 cells [36]. In the present study, GCs exhibited increased *Cyclin A1* and *B1* mRNA expression after Luman knockdown. Cyclin A1 may function as the M-phase cyclin since the cell cycle progression was arrested at S phase in cyclin A1-deficient mice [37]. Cyclin B1 is associated with cyclin-dependent kinase 1 (cdk1), and is the key regulators of cell cycle progression from S phase to G2/M phase [38]. Thus, we surmised that the higher mRNA expression levels of *Cyclin A1* and *Cyclin B1* may be responsible for promoting cell cycle progression from S to M phase after Luman knockdown. Moreover, the mRNA levels of *Cyclin D2* and *E* were also up-regulated after Luman knockdown. These two genes are important regulators in the promotion of cell proliferation in granulosa cells in response to mitogenic stimuli [39,40]. These results indicate that Luman is important in maintaining normal cell cycle regulation for GC growth and proliferation.

Apoptosis, a physiological form of cell death, is the cellular mechanism underlying ovarian follicular atresia. Previous studies have shown that GC apoptosis is regulated by caspases3 and Bcl-2 gene family members, including Bax and Bcl-2 [41–43]. Liang *et al*. demonstrated that Luman overexpression protects cells from ER stress-induced apoptosis [10]. Thus, we hypothesized that Luman knockdown may promote cell survival by attenuating apoptosis in mouse GCs. However, our results showed that Luman knockdown has no significant effects on apoptosis in mouse GCs, as determined via flow cytometry (Table 3). At the transcriptional level, we only observed a significant decrease of *Bcl-2* mRNA expression (Fig 4), which is considered as a key factor in the balance between the initiation and prevention of apoptosis in female germ cells [44]. The key executioner of apoptosis, Caspase 3 [45], is not significantly affected by Luman knockdown at mRNA and protein level. Previous reports have shown that p53 facilitates apoptosis [46]. The results of the present study indicate that Luman knockdown resulted in increased p53 mRNA expression, although the increase was not statistically significant. Taken together, we speculated that Luman knockdown may affect some apoptosis-related factors at mRNA level, but not be able to produce detectable percentages of apoptotic cells in a GC culture model.

In this study, the mRNA expression of *Has2* and *Ptgs2* were measured to evaluate the regulatory functions of Luman in mouse GCs. The results showed that the mRNA expression of *Has2* was decreased, while that of *Ptgs2* was increased by Luman knockdown. Hyaluronan synthase 2 (Has2) is a membrane-bound enzyme to synthesize the glycosaminoglycan hyaluronan. It is essential for cumulus expansion as its product forms matrix during cumulus expansion in response to the ovulatory LH surge [47]. The decrease of Has2 is an indication of hyaluronan shortage during cumulus expansion. Prostaglandin synthase 2 (Ptgs2) is the rate-limiting enzymes in the production of prostaglandins. It is essential for ovulation but is also required for luteinization [48]. The increase of Ptgs 2 may affect cumulus expansion and ovulation. Accordingly, we speculated that Luman may participate in folliculogenesis, ovulation, and luteinization by regulating the expression of these genes in the mouse ovary.

In conclusion, this study showed that Luman knockdown may affect mouse GC cell cycle activity, promote cell proliferation and decrease estradiol and progesterone synthesis by controlling the expression of steroidogenic genes. The roles of Luman in the regulation of folliculogenesis, ovulation, and luteal tissue formation were also demonstrated by the effects of its knockdown on gene expression in mouse GCs.
